# Public Discourse, User Reactions, and Conspiracy Theories on the X Platform About HIV Vaccines: Data Mining and Content Analysis

**DOI:** 10.2196/53375

**Published:** 2024-04-03

**Authors:** Jueman M Zhang, Yi Wang, Magali Mouton, Jixuan Zhang, Molu Shi

**Affiliations:** 1 Harrington School of Communication and Media University of Rhode Island Kingston, RI United States; 2 Department of Communication University of Louisville Louisville, KY United States; 3 School of Rehabilitation Sciences University of Ottawa Ottawa, ON Canada; 4 Polk School of Communications Long Island University Brooklyn, NY United States; 5 College of Business University of Louisville Louisville, KY United States

**Keywords:** HIV, vaccine, Twitter, X platform, infodemiology, machine learning, topic modeling, sentiment, conspiracy theory, COVID-19

## Abstract

**Background:**

The initiation of clinical trials for messenger RNA (mRNA) HIV vaccines in early 2022 revived public discussion on HIV vaccines after 3 decades of unsuccessful research. These trials followed the success of mRNA technology in COVID-19 vaccines but unfolded amid intense vaccine debates during the COVID-19 pandemic. It is crucial to gain insights into public discourse and reactions about potential new vaccines, and social media platforms such as X (formerly known as Twitter) provide important channels.

**Objective:**

Drawing from infodemiology and infoveillance research, this study investigated the patterns of public discourse and message-level drivers of user reactions on X regarding HIV vaccines by analyzing posts using machine learning algorithms. We examined how users used different post types to contribute to topics and valence and how these topics and valence influenced like and repost counts. In addition, the study identified salient aspects of HIV vaccines related to COVID-19 and prominent anti–HIV vaccine conspiracy theories through manual coding.

**Methods:**

We collected 36,424 English-language original posts about HIV vaccines on the X platform from January 1, 2022, to December 31, 2022. We used topic modeling and sentiment analysis to uncover latent topics and valence, which were subsequently analyzed across post types in cross-tabulation analyses and integrated into linear regression models to predict user reactions, specifically likes and reposts. Furthermore, we manually coded the 1000 most engaged posts about HIV and COVID-19 to uncover salient aspects of HIV vaccines related to COVID-19 and the 1000 most engaged negative posts to identify prominent anti–HIV vaccine conspiracy theories.

**Results:**

Topic modeling revealed 3 topics: HIV and COVID-19, mRNA HIV vaccine trials, and HIV vaccine and immunity. HIV and COVID-19 underscored the connections between HIV vaccines and COVID-19 vaccines, as evidenced by subtopics about their reciprocal impact on development and various comparisons. The overall valence of the posts was marginally positive. Compared to self-composed posts initiating new conversations, there was a higher proportion of HIV and COVID-19–related and negative posts among quote posts and replies, which contribute to existing conversations. The topic of mRNA HIV vaccine trials, most evident in self-composed posts, increased repost counts. Positive valence increased like and repost counts. Prominent anti–HIV vaccine conspiracy theories often falsely linked HIV vaccines to concurrent COVID-19 and other HIV-related events.

**Conclusions:**

The results highlight COVID-19 as a significant context for public discourse and reactions regarding HIV vaccines from both positive and negative perspectives. The success of mRNA COVID-19 vaccines shed a positive light on HIV vaccines. However, COVID-19 also situated HIV vaccines in a negative context, as observed in some anti–HIV vaccine conspiracy theories misleadingly connecting HIV vaccines with COVID-19. These findings have implications for public health communication strategies concerning HIV vaccines.

## Introduction

### Background

Vaccination has long been recognized as a crucial preventive measure against diseases and infections, but opposition to vaccines has endured [1]. HIV vaccination has been regarded as a potential preventive measure to help combat the HIV epidemic in the United States, with research and progress dating back to the mid-1980s but without success thus far [2]. An estimated 1.2 million people were living with HIV in the United States by the end of 2021, with 36,136 new HIV diagnoses reported in 2021 [3].

On January 27, 2022, the biotechnology company Moderna announced the initiation of clinical trials for an HIV vaccine using messenger RNA (mRNA) technology [4]. In March 2022, the National Institutes of Health announced the start of clinical trials for 3 mRNA HIV vaccines [5]. The mRNA technology had previously been used in the Pfizer-BioNTech and Moderna COVID-19 vaccines, which protected individuals against severe symptoms and fatalities during the pandemic [6]. Following the successes of mRNA COVID-19 vaccines, which led to the Nobel Prize in Physiology or Medicine being awarded to 2 scientists in October 2023 [7], researchers have been investigating the potential of mRNA vaccines for various other diseases, including HIV [8,9]. The announcements of clinical trials for mRNA HIV vaccines revived public discussion on the prospect of vaccines to combat HIV [9] despite >3 decades of unsuccessful research [2]. Meanwhile, these announcements were made against the backdrop of intense vaccine debates during the COVID-19 pandemic, with misinformation and conspiracy theories fueling vaccine hesitancy [10-12].

The X platform, formerly known as Twitter, has been a significant social media platform and a vital source for text-based public discourse. Posts on X have been studied to understand public discourse about vaccines in general [13-15] and about specific vaccines, such as COVID-19 vaccines in recent years [12,16,17]. However, there is a dearth of research about public discourse on HIV vaccines on social media. Given the advancement in mRNA technology in COVID-19 vaccines and heated vaccine debates, it has become especially important to gain insights into public discourse and reactions regarding potential new vaccines.

This study is grounded in the growing field of infodemiology and infoveillance, which investigates the “distribution and determinants of information in an electronic medium,” specifically on the web, by analyzing unstructured text with the aim of informing public health practices or serving surveillance objectives [18]. In recent infodemiology and infoveillance studies, machine learning algorithms have been increasingly used to examine substantial amounts of social media content, such as posts on X related to COVID-19 vaccines [12,16,17] and HIV prevention [19], to extract insights into public discourse and reactions. These algorithms automatically analyze extensive volumes of posts and capture latent textual information such as topics and sentiments. This study aimed to investigate how users used different post types to contribute original content to topics and valence identified through machine learning algorithms and how these topics and valence affected user reactions on X regarding HIV vaccines. In addition, by manually coding the most engaged posts, similar to an approach used in previous infodemiology and infoveillance research [20], the study intended to identify salient aspects of HIV vaccines related to COVID-19 as well as prominent anti–HIV vaccine conspiracy theories. Analyzing posts on X about HIV vaccines can shed light on public discourse and information diffusion. These findings have implications for shaping public health communication strategies about HIV vaccines [18]. Furthermore, the findings may help in understanding the acceptability of the HIV vaccine upon its successful development in comparison with adherence to existing HIV prevention measures. Previous infodemiology and infoveillance research effectively increased the forecast accuracy of COVID-19 vaccine uptake by leveraging insights derived from posts on X [21].

### Literature Review

#### Public Discourse About HIV Prevention on X

Social media platforms have become important channels for HIV communication, enabling the dissemination of and engagement with content encompassing a wide array of issues related to HIV prevention, treatment, coping, and available resources [22,23]. An earlier infodemiology study examined 69,197 posts on the X platform containing the hashtag #HIVPrevention between 2014 and 2019 and categorized these posts into 10 identified topics concerning HIV prevention [19]. Among them, pre-exposure prophylaxis had the highest representation with 13,895 posts, followed by HIV testing; condoms; harm reduction; gender equity and violence against women; voluntary medical male circumcision; sex work; postexposure prophylaxis; elimination of mother-to-child transmission of HIV; and abstinence, which had the lowest representation with 180 posts. Furthermore, that study suggested a consistency between the volume of posts related to specific HIV prevention measures on X over time and the temporal trends in the uptake of those measures [19]. It is noteworthy that the topic of HIV vaccines was absent, which suggested minimal public discourse on the topic during these years. This may be associated with the extensive history of unsuccessful research in this area [2].

Despite the availability of current HIV prevention measures, efforts have been made to develop HIV vaccines, which are considered necessary to bridge the gap between the challenges in adhering to existing HIV prevention measures and the ambitious goal set by United Nations member states to end the HIV epidemic by 2030 [24,25]. The surge in public discussion about HIV vaccines, possibly elicited by the clinical trials for mRNA HIV vaccines [9], presented an optimal opportunity to investigate public discourse and reactions regarding HIV vaccines. To the best of our knowledge, this is the first study to analyze posts on X about HIV vaccines.

#### Public Discourse and Post Types on X

On the X platform, public discourse featuring original content can be observed through 3 post types: self-composed posts, quote posts, and replies [26]. X users can compose a post. They can also create a quote post, which entails reposting a post while adding their comments. In addition, they can reply to a post to share their comments [26]. While self-composed posts initiate new conversations, quote posts and replies enable users to join existing conversations by contributing their own comments [27]. The Pew Research Center’s analysis of survey respondents’ posts on X from October 2022 to April 2023 revealed the composition of different types of posts. Regarding the 3 types of posts containing original content, replies accounted for the highest proportion at 40%, followed by self-composed posts at 15% and quote posts at 9%. The remaining 35% were reposts [28].

Machine learning algorithms have been increasingly used in recent years to identify latent message features, including textual topics and sentiment valence, among vast numbers of social media posts, as exemplified by previous research analyzing posts on X about COVID-19 vaccines [12,16,17] and HIV prevention [19]. However, the patterns of public discourse in social media conversations are unclear. Specifically, there is a scarcity of research on how people contribute their original content to topics and valence related to a public health issue. This study aimed to address this gap by examining the relationship between post types and message features, specifically topics and valence uncovered using machine learning algorithms, with a focus on HIV vaccines as the subject matter. The findings will advance our knowledge of user contributions to social media conversations about HIV vaccines.

#### Message Features Influencing User Reactions on X

Examining message diffusion on social media has been a multifaceted challenge, especially with vaccines being a contentious issue debated fervently during the COVID-19 pandemic [16]. Another contribution of this study is to advance this research area by using machine learning to investigate the synergistic impact of content and account features on user reactions regarding a potential new vaccine amid the context of intense vaccine debates.

The extent to which a message results in optimal diffusion on social media can be gauged by user reactions [16,29-31]. On X, a user can engage with posts—be it a self-composed post, quote post, or reply—in 2 primary 1-click reactions: liking and reposting [26]. An X user can like a post to show appreciation for it or repost it to share it publicly. Compared to liking, reposting is a more social behavior [16,32]. Unlike X’s old timeline, which mostly displayed posts from accounts that a user followed, its current “For you” timeline also shows posts that those accounts have engaged with along with other posts recommended based on user reactions [33]. The nature of promoting posts based on user reactions makes it more important to investigate the factors that influence user reactions.

This study investigated 2 categories of message-level features that, according to previous research, can drive user interactions: content features in terms of topics and valence and account features in terms of user verification and follower count. Post topics affect likes and reposts on X [16,30,34]. Previous research on COVID-19 vaccine posts on X has indicated that posts containing useful information garner more likes and reposts [16]. This is likely because information utility fills people’s knowledge gaps and serves their utilitarian needs in the face of health risks [16,32,34-36]. In addition, previous studies have suggested that the novelty of useful information further facilitates sharing of digital health information [32,36], such as updates about COVID-19 vaccine development [12]. Given the initial success of mRNA technology in COVID-19 vaccines, mRNA HIV vaccine candidates may possess the inherent features of prospective usefulness and ongoing novelty. As a result, posts presenting pertinent information have the potential to generate more likes and reposts. Meanwhile, the announcements of clinical trials for mRNA HIV vaccines were made amidst intense vaccine debates during the COVID-19 pandemic [12]. Previous research has shown that perceived controversiality in health information increases viewership but not sharing on social media [32]. In the context of the heated controversy surrounding vaccines, it is crucial to understand user reactions to new potential vaccines.

In addition to post topics, post valence can play a role in user reactions [34]. Past research has generally revealed that there are more positive than negative posts on X about vaccines in general [13-15] and, more recently, about COVID-19 vaccines in particular [12,16,17]. However, the influence of post valence on user reactions remains unclear. One study on COVID-19 vaccines showed that positive posts on X received more likes but not more reposts [16]. Another study on vaccines regardless of their type revealed that antivaccine posts garnered more reposts than provaccine posts on X [13]. A psychological rationale supporting the social transmission of positive content is the motivation of individuals to present themselves positively and shape their self-identity [35,37]. In comparison, social transmission of negative content can be attributed to the idea that certain negative content triggers activation, which drives user reactions [35].

Furthermore, previous research has shown that account features such as verification status and follower count affect user reactions on social media [13,16,34]. Given the vast amounts of information available in the digital age, the authenticity of user accounts becomes crucial in the diffusion of health information. One study revealed that account verification enhanced the number of likes and reposts for posts about COVID-19 vaccines on X [16]. Another study indicated that follower counts increased the number of reposts for posts about vaccines on X regardless of vaccine type [13].

#### Conspiracy Theories

A conspiracy theory refers to the belief that a coalition of powerholders forms secret agreements with malevolent intentions [38,39]. It differs from other types of misinformation by hypothesizing a pattern in which people, objects, or events are interconnected in a causal manner [39]. Previous research has revealed conspiracy theories as a salient theme in antivaccine discourse on social media, along with other themes such as side effects and inefficacy [40,41]. For HIV vaccines, conspiracy theories are crucial in understanding public discourse against them given the limited information about side effects and inefficacy until future success. An additional contribution of this study is the identification of prominent anti–HIV vaccine conspiracy theories through manual coding of the most engaged with negative posts.

Antivaccine conspiracy theories contribute to vaccine hesitancy [42-44], as observed recently with COVID-19 vaccines [10,11]. Understanding the themes and reasoning behind antivaccine conspiracy theories will provide vital implications for deploying evidence-based and logic-driven strategies to counter them [45-47]. A systematic review of antivaccine discourse on social media from 2015 to 2019 revealed pre–COVID-19 conspiracy theories [41]. These theories claimed that powerholders promoted vaccines for self-serving interests, including hiding vaccine side effects for financial gain and controlling society and the population [40,41]. During the COVID-19 pandemic, antivaccine conspiracy theories thrived on social media. Some theories claimed that the pandemic was invented for pharmaceutical companies’ profit from vaccines [44], whereas others linked mRNA COVID-19 vaccines to infertility and population control [10,11,44,48,49]. Another conspiracy theory claimed that Bill Gates and the US government aimed to implant trackable microchips into people through mass vaccination [11,27,49]. This aligns with conspiracy theories from earlier years. In particular, the Big Pharma conspiracy theory claims that pharmaceutical companies, together with politicians and other powerholders, conspire against the public interest [50]. The New World Order conspiracy theory alleges that a power elite with a globalization agenda colludes to rule the world [51]. Conspiracy theories have also linked other vaccines, such as poliovirus vaccines in the past [52,53] and COVID-19 vaccines in recent years, to HIV infection [54,55]. These conspiracy theories were based on the claims that alleged vaccines contained HIV.

### Research Questions

To understand public discourse and reactions surrounding HIV vaccines on the X platform, we put forward the following research questions (RQs):

1. What are the topics of the posts about HIV vaccines? (RQ 1)
2. What is the valence of the posts about HIV vaccines? (RQ 2)
3. How do topics and valence vary across different types of posts? (RQ 3)
4. How do content features (topics and valence) and account features (verification status and follower count) affect 1-click reactions in terms of likes and reposts, respectively? (RQ 4)
5. What are the prominent anti–HIV vaccine conspiracy theories that receive the most reactions? (RQ 5)

## Methods

### Data Source

We collected English-language original posts about HIV vaccines on the X platform from January 1, 2022, to December 31, 2022, using Netlytic [56]. The selected time frame began in January 2022 with the initiation of mRNA HIV vaccine clinical trials fueling public discussion and concluded in December 2022, a significant month for HIV and AIDS awareness marked by World AIDS Day on the first day of the month. Posts, excluding reposts, that contained both keywords (case insensitive)—“HIV” and “vaccine”—were extracted, resulting in a total of 36,424 posts across 365 days. Posts were collected weekly. Posts published from the last ending time point to at least 24 hours before each collection time point were included in the data set, allowing for a substantial reaction time.

### Procedure

The unit of analysis was a post. For each post, automated extraction produced data for user reactions (the number of likes and reposts) as well as account features (account verification status and follower count). All 36,424 posts underwent topic modeling using latent Dirichlet allocation (LDA) to identify latent topics, as well as sentiment analysis using Valence Aware Dictionary and Sentiment Reasoner (VADER) to access valence. LDA generated topic-specific loadings and identified the dominant topic for each post. VADER generated a valence compound score for each post, which was also categorized as positive, neutral, or negative based on standard VADER classification values.

LDA revealed 3 topics. As the topic of HIV and COVID-19 dominated in a large proportion of posts, we manually coded the 1000 most engaged posts containing the words “HIV” and “COVID” to uncover the salient aspects of HIV vaccines related to COVID-19. To develop coding for subtopics, 2 researchers initially reviewed and coded the top 200 posts with the most reactions. Subtopics were categorized by adapting existing categories from the literature [16,34] and integrating newly identified subtopics from the posts. The Scott π was 0.80 for categorizing subtopics. Subsequently, each researcher independently coded half of the remaining 800 posts.

We then conducted cross-tabulation analyses among all posts to examine the distribution of topics and valence among different types of posts. Furthermore, we conducted linear regression analyses among all posts to assess the influence of content and account features on these 1-click reactions. Of all 36,424 posts, 19,284 (52.94%) received ≥1 like, and 9155 (25.13%) received ≥1 repost. We added a constant value of 1 to all data points for likes and reposts before applying the natural logarithm. This was done to include posts with 0 likes or reposts and to mitigate the skewness of the data distribution.

Of the 28,439 posts that received likes or reposts, 6176 (21.72%) were negative. We manually coded the top 1000 negative posts with the most reactions to uncover prominent anti–HIV vaccine conspiracy theories. To develop coding for conspiracy theories, 2 researchers initially reviewed and coded the top 200 negative posts that received the most reactions. Posts containing conspiracy theories were identified based on expressions of postulated causal connections between people, objects, or events with malevolent intent [38,39]. Conspiracy theories were then classified based on the existing ones from the literature [50,51] and the emerging ones observed in the posts. Coding discrepancies were resolved through a further review of questionable posts and refinement of the conspiracy theories following the approach used in previous social media content analyses [40,57]. The procedure identified conspiracy theories and established intercoder reliability. The Scott π was 0.83 for identifying conspiracy theories and 0.81 for categorizing them. Each researcher then independently coded half of the remaining 800 negative posts.

### Measures

#### User Reactions

One-click reactions were measured by the number of likes and reposts, which were automatically extracted. Because a small number of posts garnered significant 1-click reactions, the distribution of likes and reposts was right skewed. To reduce right skewness, we used the natural logarithm of the number of likes and reposts in linear regression analyses, as done in previous research [16,30,34].

#### Post Topics

All posts underwent topic modeling using LDA [58]. Topic modeling is a commonly used unsupervised learning method that generates a probabilistic model for a corpus of text data [59]. As a widely used topic model [59], LDA has been applied to discover topics within rich sources of digital health information, such as electronic health records [60], reviews on the web [61], and posts on X [16,34].

LDA relies on 2 matrices to define the underlying topical structure: the word-topic matrix and the document-topic matrix [62]. In this study, a post was considered a document. The general idea is that a post is represented by a Dirichlet distribution of latent topics, with each latent topic being represented by a Dirichlet distribution of words [59]. In the word-topic matrix, where the rows represent words and the columns represent topics, each element reveals the conditional probability of a word appearing within a topic [62]. A topic can be interpreted by examining a list of the most probable words ranked by their frequencies within a given topic using 3 to 30 words [63]. In the document-topic matrix, where rows represent posts and columns represent topics, each element reveals the conditional probability of a topic underlying a post [62]. In other words, it reveals the topic-specific loadings for each post.

When interpreting each topic, we reviewed the word-topic matrix as well as sample posts with high topic-specific loadings and significant reactions. LDA generated topic-specific loadings for each post ranging from 0 to 1, with values closer to 1 indicating a higher probability of a topic being associated with a post. Furthermore, LDA determined the dominant topic for each post by selecting the topic with the highest topic-specific loading among all topics. In the cross-tabulation analysis examining the distribution of topics across post types, the dominant topic for each post was entered for analysis. In the linear regression models assessing message-level drivers of user reactions, topic-specific loadings for each post were entered as topic values following previous research [16,34].

#### Post Valence

We used VADER to analyze the sentiment valence of each post. VADER is a rule-based model specifically attuned for assessing sentiments expressed in social media text [64]. VADER generated a compound valence score for each post ranging from –1 to 1, with a value of –1 indicating the most negative sentiment and a value of 1 indicating the most positive sentiment [65]. The standard VADER compound value thresholds for classifying valence categories are as follows: 0.05 to 1 for positive, −0.05 to 0.05 for neutral, and −0.05 to −1 for negative [65]. In the cross-tabulation analysis examining the distribution of valence among post types, the valence category for each post was entered for analysis. In the linear regression models assessing message-level drivers of user reactions, the VADER compound valence score for each post was used.

#### Post Type

This study collected original posts excluding reposts. For each original post, it was automatically extracted whether it was a self-composed post, a quote post with comments, or a reply.

#### Conspiracy Theories

In total, 2 researchers manually coded the top 1000 out of 6176 negative posts with the highest total number of likes and reposts to uncover highly engaged conspiracy theories. They distinguished conspiracy theories from other types of negative information, particularly other types of misinformation, by recognizing the presence of a hypothesized pattern of causal connections between people, objects, or events for malicious intent [38,39]. Conspiracy theories were then categorized based on the existing ones from the literature and the emerging ones observed in the posts.

As an example, consider a post paraphrased as follows:

Image using condoms consistently, only to contract HIV from a COVID vaccine.

It was posted on February 9, 2022, and received 783 likes and 296 reposts. This post was not coded as displaying a conspiracy theory as it only presented misinformation suggesting that COVID-19 vaccines caused HIV. In comparison, another post was paraphrased as follows:

The COVID vaccine contained a spike protein derived from HIV. I was banned from saying this and ridiculed for months. Also, pharmacies stock up HIV self-tests.

It was posted on February 8, 2022, with 147 likes and 48 reposts. This post was coded as displaying a conspiracy theory. It was further classified within the category of conspiracy theories linked to COVID-19 vaccines containing, causing, or increasing HIV. This post suggested a hypothesized pattern of maliciously intended causal connections between the claim that the COVID-19 vaccine contained HIV and the stocking of HIV self-tests in pharmacies. As another example, a post was paraphrased as follows:

Scientists uncover a “highly virulent” strain of HIV in the Netherlands.

It was posted on February 12, 2022, and received 11 likes and 11 reposts. This post conveyed negative information but did not present a conspiracy theory. In comparison, another post was paraphrased as follows:

By coincidence again, the development of a new mRNA HIV vaccine began just before the emergence of the new HIV strain.

It was posted on February 8, 2022, and received 102 likes and 4 reposts. This post was coded as presenting a conspiracy theory and further classified into the category of conspiracy theories linked to the identification of a new highly virulent HIV strain. This post emphasized the speculative timing of the discovery of the new highly virulent HIV strain occurring shortly after the announcement of the development of a new mRNA HIV vaccine.

#### Account Features

For each post, the posting account’s verification status and follower count were automatically extracted.

### Data Analysis

We used cross-tabulation analyses to investigate the distribution of topics and valence across different post types, in which the dominant topic and valence category for each post were entered, respectively, alongside the post type. We used linear regression models to examine the message-level drivers of user reactions among posts that received likes or reposts. In the linear regression models, a constant value of 1 was added to all data points of like and repost counts. The natural log-transformed values for each post were then regressed on 3 topic-specific loadings generated from LDA, the valence compound score generated from VADER, and 2 autoextracted account features—account verification status and follower count. The “plus one” technique was used to include posts that received 0 likes or reposts and to address the skewness of the data distribution.

### Ethical Considerations

Following Long Island University’s institutional review board determination process, an institutional review board review was deemed unnecessary for this study, which collected and analyzed publicly available social media data. All referenced posts were paraphrased to avoid association with any particular user on the X platform.

## Results

### Post Topics

RQ 1 asked about the topics present in all the posts. We trained a topic model using LDA exploring topic numbers ranging from 2 to 20. The optimal number of topics (*k*) was selected considering both the coherence score (*C_v_*) and the topic model visualization in a Python library called *pyLDAvis*, as done in previous research [16,66]. *C_v_* is a metric that reflects the semantic coherence of topics by evaluating the word co-occurrence likelihood within topics [67]. A higher *C_v_* indicates a better classification achieved by the topic model. In this study, the model with 2 topics (*k*=2) yielded the highest *C_v_* (0.42), whereas the model with 3 topics (*k*=3) yielded the second highest *C_v_* (0.35). The *pyLDAvis* chart depicts each topic as a circle. Overlapping areas between circles suggest similarities in topics. Thus, a chart without overlapping circles is preferable for *k*. The *pyLDAvis* chart for this study showed that, when the value of *k* was 2 or 3, the circles did not overlap. However, when *k* reached 4, the circles began to overlap, and overlapping circles persisted for values of *k* ranging from 4 to 20. Between the *k* values of 2 and 3, we opted for a model comprising 3 topics (*k*=3) considering that a smaller number of topics tends to result in overly broad meanings for each topic [68].

Table 1 summarizes the 3 topics and lists their representative posts. Each topic was interpreted by examining the top 10 probable words ranked by frequency, along with sample posts exhibiting high topic-specific loadings and 1-click reactions. Topic 1 was HIV and COVID-19, covering 78% of the tokens [69] and dominating in 92.46% (33,678/36,424) of the posts. Topic 2 was mRNA HIV vaccine trials, covering 14% of the tokens and dominating in 5.91% (2151/36,424) of the posts. Topic 3 was HIV vaccine and immunity, covering 8% of the tokens and dominating in 1.63% (595/36,424) of the posts.

**Figure 1:**
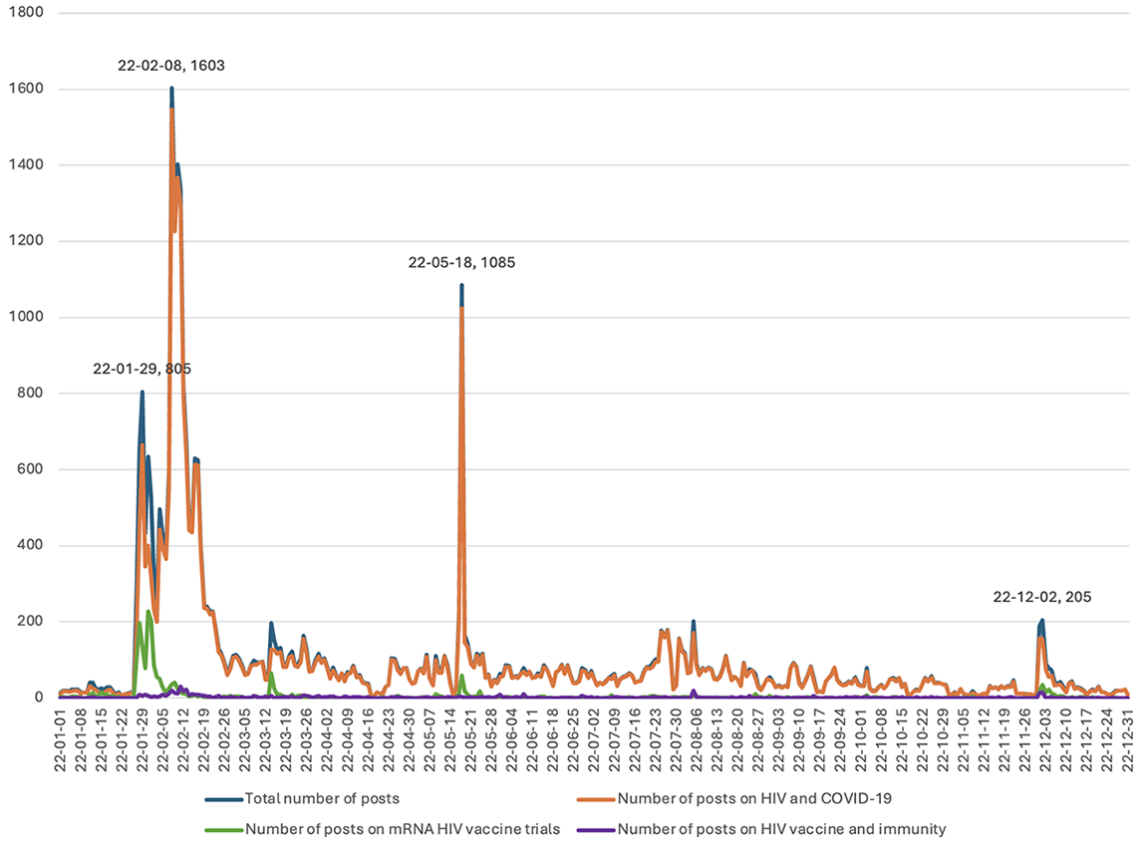
Number of original posts about HIV vaccines in total and by topic in 2022. mRNA: messenger RNA.

Figure 1 illustrates the daily numbers of original posts about HIV vaccines throughout 2022, in total and categorized into 3 topics. Moderna’s announcement of clinical trials for its first mRNA HIV vaccine on January 27, 2022, likely triggered the initial surge, culminating in a daily peak when the number of posts reached 805 on January 29, 2022. The daily number of posts about mRNA HIV vaccine trials (topic 2) in the week following Moderna’s announcement was higher than on other days throughout the year. Nevertheless, even during that week, there were higher daily numbers of posts about HIV and COVID-19 (topic 1), which remained dominant among the 3 topics during the entire year. The year’s second and highest daily peak occurred on February 8, 2022, recording a total of 1603 posts, most of which focused on HIV and COVID-19 (topic 1). This could be attributed to the emergence of new HIV-related events in early February 2022, including the promotion of HIV tests by public figures [64] and the discovery of a new highly virulent HIV strain [65]. The third highest daily peak, comprising 1085 posts, occurred on May 18, 2022, which has marked HIV Vaccine Awareness Day since 1998. Most of the posts centered on HIV and COVID-19 (topic 1). The remainder of the year did not reach such high peaks, with the largest daily volume of 205 posts occurring on December 2, 2022, the day following World AIDS Day, observed since 1988. Similar to previous daily peaks, most of the posts revolved around HIV and COVID-19 (topic 1).

The results revealed the dominance of HIV and COVID-19 (topic 1) in 92.46% (33,678/36,424) of the posts, with *HIV* as the most frequent word and *COVID* as the fourth most frequent word. To gain a deeper understanding of salient aspects of HIV vaccines related to COVID-19, we manually coded the top 1000 posts with the highest total number of likes and reposts that contained both *HIV* and *COVID*. Table 2 summarizes the subtopics and their representative posts with like and repost counts.

The first major subtopic, comprising 24% (240/1000) of the posts, focused on the reciprocal influence of HIV vaccines and COVID-19 vaccines on each other’s development. Years of HIV vaccine research facilitated the rapid development of mRNA COVID-19 vaccines, and the success of COVID-19 vaccines might accelerate the development of mRNA HIV vaccines. The second major subtopic, comprising 17.6% (176/1000) of the posts, involved comparisons between HIV and COVID-19 in various aspects. Specifically, the development speed of HIV vaccines compared to COVID-19 vaccines was a major point of comparison. In addition, some posts questioned whether potential HIV vaccines could be comparable to COVID-19 vaccines in terms of cost and accessibility during rollout. Others raised concerns about efficacy, safety, and inequality for both vaccines. The third major subtopic, comprising 26.5% (265/1000) of the posts, connected COVID-19 vaccines with HIV. One issue discussed was whether COVID-19 vaccines contained, caused, or increased HIV. Another issue raised was distinguishing between HIV symptoms and COVID-19 vaccine side effects, such as a fabricated condition called *VAIDS*, short for vaccine-acquired immunodeficiency syndrome. The fourth major subtopic, comprising 13.6% (136/1000) of the posts, featured conspiracy theories that presented hypothesized patterns linking COVID-19, HIV, and their vaccines with malicious intent. Prominent conspiracy theories in this subtopic included connecting misinformation that COVID-19 vaccines contain, cause, or increase HIV with the ongoing development of HIV vaccines; associating HIV and AIDS symptoms with side effects of COVID-19 vaccines; and claiming that COVID-19 originated from unsuccessful HIV vaccine research. As this study also manually coded the 1000 most engaged negative posts to identify prominent conspiracy theories, additional results pertaining to conspiracy theories will be discussed further in another subsection. The remaining posts related to HIV and COVID-19 included those that generally mentioned research on them or made connections without specifying details.

**Table 1:** Summary of topics with representative paraphrased posts (N=36,424).

Topic number and label	Top 10 words by frequency	Topic proportion (% of tokens)	Posts, n (%)	Valence, mean (SD)	Representative paraphrased posts
Topic 1: HIV and COVID-19	Vaccine HIV Use Covid Aids Year Get People Test Make	77.9	33,678 (92.46)	0.055 (0.480)	It’s astonishing that some people are opposed to vaccinations, when COVID vaccines have paved the way for HIV vaccine trials. (January 29, 2022; topic-specific loading: 0.862; likes: 30,591; reposts: 370) Let me clarify. A small portion of the HIV virus was added in the Covid vaccine as to stabilize the protein. This vaccine was given to people. Now, a year later, they are indicating a rise in HIV cases, urging people to get HIV tests while developing an HIV vaccine. (February 8, 2022; topic-specific loading: 0.807; likes: 3207; reposts: 1456)
Topic 2: mRNA^a^ HIV vaccine trials	Trial mRNA Moderna Technology Clinical Begin Experimental First Launch News	14.4	2151 (5.91)	0.040 (0.363)	I am thrilled to share exciting news. The first patient has received a dose in the phase 1 trial of Moderna’s mRNA-1644, an HIV vaccine candidate, which utilizes the same mRNA technology as our COVID-19 vaccines! (January 27, 2022; topic-specific loading: 0.521; likes: 18,895; reposts: 881) I have some exciting news to share. The first patient has received a dose in the phase 1 trial of Moderna’s and National Institutes of Health HIV trimer vaccine candidate, mRNA-1574, which utilizes the same mRNA technology as our COVID-19 vaccines! (March 21, 2022; topic-specific loading: 0.585; likes: 7056; reposts: 1224)
Topic 3: HIV vaccine and immunity	Response Immune Risk Protein Antibody Immunity Spike_protein High Add GT	7.8	595 (1.63)	−0.008 (0.445)	New study: Analyzing two cooperating antibodies reveals immune pressure on HIV Env to elicit a V3-glycan supersite broadly neutralizing antibody lineage. The goal of vaccine is to stimulate broadly neutralizing antibodies. (September 26, 2022; topic-specific loading: 0.602; likes: 11; reposts: 4) QTNSPRRAR is the t-cell CD8 activator essential in establishing a durable t-cell response to ACE2 receptor binding. This creates a sustained t-cell response to binding and then the antibody response to HIV like proteins, which is like all other t-cell vaccine adjuvants. (April 25, 2022; topic-specific loading: 0.506; likes: 12; reposts: 1)

^a^mRNA: messenger RNA.

**Table 2:** Subtopics of HIV and COVID-19 and representative posts with a high number of reactions (N=1000).

Subtopic			Posts, n (%)		Paraphrased posts with a high number of reactions in each subtopic		Reaction count^a^ (likes and reposts)
**Reciprocal impact (n=240)**							
	COVID-19 vaccine research aiding HIV vaccines	211 (87.9)		The progress of items like PrEP^b^ shouldn’t be underestimated. The medical advancements with COVID boosted HIV vaccine development. The future is promising. (January 28, 2022)		4342 (4100 and 242)	
	HIV vaccine research aiding COVID-19 vaccines	29 (12.1)		Great presentation, addressing HIV and COVID vaccines. Despite 40 years of dedicated work, an HIV vaccine remains elusive, but the work was crucial in developing effective COVID vaccines. (February 13, 2022)		80 (62 and 18)	
**Comparisons (n=176)**							
	HIV and COVID-19 vaccines	61 (34.7)		Will this new HIV “vax” work similarly to the COVID vaccine? Does it not prevent transmission, acquisition, and have a short duration of efficacy? “It’s just a slight case of AIDS”? (February 16, 2022)		225 (173 and 52)	
	Development speed of HIV vs COVID-19 vaccines	32 (18.2)		HIV was first identified 40 years ago, yet we still lack a vaccine, with only 3 cures. COVID emerged just over 2 years ago, and highly effective vaccines have saved over a million American lives. (February 16, 2022)		3058 (2710 and 348)	
	Vaccine inequality	19 (10.8)		Ready for the widespread distribution of an HIV vaccine? We are not prepared, considering the experience of the COVID vaccine rollout. It's crucial to plan for equal access to a future “people’s vaccine.” #HIVVaccineAwarenessDay (May 18, 2022)		31 (23 and 8)	
	HIV and COVID-19 pandemics	34 (19.3)		HIV+ kids were medically neglected and lost their lives in the late ’90s, even as adults benefited from life-saving ART. Now the same situation applies to kids with COVID. No paxlovid or sotrovimab for kids under 12, and no vaccine for kids under 5. Over 1200 kids have died. Regulations are set. FDA, it’s time to act. (February 16, 2022)		3210 (2579 and 631)	
	HIV and COVID-19 symptoms	15 (8.5)		People not seroconverting still develop #LongCOVID. An initial weak immune response could result in viral persistence, as is seen with HIV. A higher pathogen load tends to lead to more severe outcomes. Earlier vaccine or therapy tend to yield better outcomes. (February 15, 2022)		90 (67 and 23)	
	Rights of the COVID-19 unvaccinated vs HIV-positive individuals	15 (8.5)		HIV positive individuals can join the military, but current service members are being discharged for refusing a COVID vaccine, even though the military has invested a significant amount of money on them. (September 27, 2022)		250 (181 and 69)	
**COVID-19 vaccines and HIV (n=265)**							
	COVID-19 vaccines containing, causing, or increasing HIV	185 (69.8)		There’s talk about the COVID vaccine potentially causing HIV. Shouldn’t those vaccinated still practice safe sex? (February 6, 2022)		439 (382 and 57)	
	HIV or COVID-19 injuries such as VAIDS^c^	31 (11.7)		Is the next big thing vaccine-induced AIDS, which is also known as COVID vaccines? (February 9, 2022)		281 (176 and 105)	
	COVID-19 vaccines for HIV-positive individuals	49 (18.5)		Sputnik V is the world’s first COVID vaccine to proven effective in HIV patients (March 29, 2022)		206 (169 and 17)	
**Conspiracy theories (n=136)**							
	Linked to COVID-19 vaccines containing, causing, or increasing HIV	69 (50.7)		Yesterday: Certain COVID vaccines might raise the risk of HIV infection. Today: Trials have begun for an HIV vaccine. (February 8, 2022)		5255 (3822 and 1433)	
	Linked to HIV or COVID-19 vaccine injuries such as VAIDS	9 (6.6)		Testing positive for HIV antibodies, as a response to a COVID vaccine containing HIV proteins, or its more severe and unexpected consequence, doesn't imply you have HIV. But it provides a convenient cover for vaccine injuries. Blame it on HIV! (February 11, 2022)		43 (36 and 7)	
	HIV vaccine research causing COVID-19	4 (2.9)		Why are COVID patients treated with an HIV medication? COVID-19 has been involved in HIV vaccine studies, and somehow components of the HIV envelope have found their way into SARS-CoV-2, resulting in coronavirus-HIV hybrids. (August 14, 2022)		47 (32 and 15)	
	Other conspiracy theories	54 (39.7)		—^d^		—	
Debunking conspiracy theories (n=31)			31 (3.1)		How antivaxxers believe time travel works so that a covid vaccine that was never distributed mystically caused a new HIV strain in 1992. (8 Feb 2022)		31 (29 and 2)
Research on COVID-19 and HIV (n=31)			31 (3.1)		Exited to work as a researcher at the intersection of TB, HIV and COVID prevention science, global health equity, and clinical ID. (June 2, 2022)		86 (83 and 3)
COVID-19, HIV, and vaccines (n=12)			12 (1.2)		The connection between COVID, COVID vaccine, HIV and AIDS. [an article link]. (May 16, 2022)		67 (32 and 35)
Other (n=109)			109 (10.9)		—		—

^a^The reaction count is the total number of likes and reposts.

^b^PrEP: pre-exposure prophylaxis.

^c^VAIDS: vaccine-acquired immunodeficiency syndrome.

^d^The categories labeled as “other” contain various topics. Thus, no representative post is displayed.

### Post Valence

RQ 2 asked about the sentiment valence present in all the posts. According to the standard VADER classification values, valence is categorized by compound scores as follows: positive (0.05 to 1), neutral (−0.05 to 0.05), and negative (−0.05 to −1) [65]. On average, all posts had a marginally positive score of 0.053. HIV and COVID-19 (topic 1) had a slightly positive average score of 0.055. The mRNA HIV vaccine trials (topic 2) had a neutral average score of 0.040, leaning toward the positive side. HIV vaccine and immunity (topic 3) had a more neutral average score of −0.0008. Moreover, 42.78% (15,584/36,424) of the posts were positive, 25.64% (9338/36,424) of the posts were neutral, and 31.58% (11,502/36,424) of the posts were negative.

### Topics and Valence Across Post Types

Of the 36,424 posts, 18,580 (51.01%) were replies, making up over half of the overall count. Self-composed posts totaled 41.6% (15,151/36,424), whereas the remaining 7.39% (2693/36,424) were quote posts. RQ 3 asked about the distribution of topics and valence among the 3 post types. As Table 3 shows, the distribution of topics varied by post type (N=36,424, χ^2^_4_=2511.4, *P*<.001). Of the self-composed posts, 85.36% (12,933/15,151) focused on HIV and COVID-19 (topic 1) and 13.21% (2001/15,151) focused on mRNA HIV vaccine trials (topic 2). In comparison, quote posts and replies exhibited a different pattern, in each case >97% of posts centering on HIV and COVID-19 (topic 1; 2616/2693, 97.14% and 18,129/18,580, 97.57%, respectively).

As Table 4 shows, the distribution of valence also varied by post type (N=36,424, χ^2^_4_=911.7, *P*<.001). The proportion of positive posts was slightly higher among self-composed posts at 44.95% (6810/15,151) compared to replies at 41.09% (7634/18,580) and quote posts at 42.33% (1140/2693). Self-composed posts had a smaller proportion of negative posts at 23.56% (3570/15,151) compared to replies at 37.64% (6994/18,580) and quote posts at 34.83% (938/2693). The proportion of neutral posts was larger for self-composed posts at 31.49% (4771/15,151) compared to quote posts at 22.84% (615/2693) and replies at 21.27% (3952/18,580).

Regarding the distribution of topics and valence among the 3 types of posts, quote posts and replies displayed similarities, whereas self-composed posts diverged. Compared to self-composed posts, which initiate new conversations, there was a higher proportion of HIV and COVID-19-related posts (topic 1) and a greater proportion of negative posts among quote posts and replies, which contribute to existing conversations.

**Table 3:** Cross-tabulation of topics across post types (N=36,424)^a^.

Topic number and label	Self-composed posts (n=15,151), n (%)	Quote posts with comments (n=2693), n (%)	Replies (n=18,580), n (%)	Total in topic (N=36,424), n (%)
Topic 1: HIV and COVID-19	12,933 (85.36)	2616 (97.14)	18,129 (97.57)	33,678 (92.46)
Topic 2: mRNA^b^ HIV vaccine trials	2001 (13.21)	52 (1.93)	98 (0.53)	2151 (5.91)
Topic 3: HIV vaccine and immunity	217 (1.43)	25 (0.93)	353 (1.9)	595 (1.63)
Total in post type (N=36,424)	15,151 (41.6)	2693 (7.39)	18,580 (51.01)	36,424 (100)

^a^N=36,424, *χ*^2^_4_=2511.4, *P*<.001.

^b^mRNA: messenger RNA.

**Table 4:** Cross-tabulation of valence across post types (N=36,424)^a^.

Valence	Self-composed posts (n=15,151), n (%)	Quote posts with comments (n=2693), n (%)	Replies (n=18,580), n (%)	Total in valence (N=36,424), n (%)
Positive	6810 (44.95)	1140 (42.33)	7634 (41.09)	15,584 (42.78)
Neutral	4771 (31.49)	615 (22.84)	3952 (21.27)	9338 (25.64)
Negative	3570 (23.56)	938 (34.83)	6994 (37.64)	11,502 (31.58)
Total in post type (N=36,424)	15,151 (41.6)	2693 (7.39)	18,580 (51.01)	36,424 (100)

^a^N=36,424, *X*^2^_4_=911.7, *P*<.001.

### Content and Account Features Influencing User Reactions

RQ 4 asked about the influence of content and account features on likes and reposts.

Liking is more common than reposting. While 52.94% (19,284/36,424) of posts received an average of 24.83 likes, ranging from 1 to 102,843, a total of 25.13% (9155/36,424) posts received an average of 11.38 reposts, ranging from 1 to 10,572. Table 5 reveals the influence of content features (topics and valence) and account features (verification status and follower count) on the natural log-transformed number of likes and reposts. Both linear regression models were significant at *P*<.001. The adjusted *R^2^* was 0.072 for the like model and 0.090 for the repost model.

Among the 3 topics identified using LDA, HIV and COVID-19 (topic 1) did not affect like counts but decreased repost counts. In comparison, mRNA HIV vaccine trials (topic 2) decreased like counts while increasing repost counts. Positive valence increased like and repost counts. Account verification status and follower count increased like and repost counts.

**Table 5:** Linear regression models on predictors of likes and reposts (N=36,424).

Variables			Logarithm				
			Like count+1^a,b^			Repost count+1^a,c^	
			Standard β	*P* value	Standard β		*P* value
**Content features**							
	**Post topic**						
		Topic 1: HIV and COVID-19	−.003	.68	−.042		<.001
		Topic 2: mRNA^d^ HIV vaccine trials	−.039	<.001	.018		.02
		Topic 3: HIV vaccine and immunity	−.910^e^	<.001	−.960^e^		<.001
	Post valence		.034	<.001	.033		<.001
**Account features**							
	Account verification status		.234	<.001	.239		<.001
	Follower count		.095	<.001	.114		<.001

^a^The natural logarithm, ln (Y_i_+1), was calculated on like and repost counts. This transformation was conducted to include posts receiving 0 likes and reposts, as well as to account for the skewness of the data distribution.

^b^*F* (model significance): *P*<.001; adjusted *R*^2^=0.072.

^c^*F* (model significance): *P*<.001; adjusted *R*^2^=0.090.

^d^mRNA: messenger RNA.

^e^The models excluded topic 3 on HIV vaccine and immunity to address multicollinearity issues arising from its correlations with topics 1 and 2. The reported standard β for topic 3 represents a possible β value if it had been included in the models.

### Posts With Most Reactions

Table 6 summarizes posts ranked within the top 5 for the number of likes and reposts presented in chronological order. It is worth noting that all posts in the top 5 for likes and reposts were self-composed. One particular post, which garnered the most likes (n=102,843) and reposts (n=10,572), expressed the incredible feeling of witnessing the development of an HIV vaccine within our lifetimes. It was posted by an unverified account on January 28, 2022, the day after Moderna’s announcement of clinical trials for its first mRNA HIV vaccine.

**Table 6:** Posts ranked in the top 5 for likes and reposts, paraphrased and in chronological order^a^.

Paraphrased posts in chronological order	Like count and rank	Repost count and rank
I am thrilled to share exciting news. The first patient has received a dose in the phase 1 trial of Moderna’s mRNA-1644, an HIV vaccine candidate, which utilizes the same mRNA technology as our COVID-19 vaccines! (January 27, 2022)	18,895; third	10,572; first
We are witnessing the development of an HIV vaccine within our lifetimes. How amazing is that? (January 28, 2022)	102,843; first	3881; fourth
It’s astonishing that some people are opposed to vaccinations, when Covid vaccines have paved the way for HIV vaccine trials. (January 29, 2022)	32,115; second	4370; second
I have some exciting news to share. The first patient has received a dose in the phase 1 trial of Moderna’s and National Institutes of Health HIV trimer vaccine candidate, mRNA-1574, which utilizes the same mRNA technology as our COVID-19 vaccines! (March 21, 2022)	7056; fifth	1224
Many medical and pharmaceutical achievements including HIV vaccine in progress. What an incredible time to be living in!” (June 3, 2022)	5034	2144; fifth
I am overjoyed to share exciting news. Despite being in an early phase 1 trial, results for HIV vaccine candidate eOD-GT8 60mer have revealed 97% of participants (with only one exception) produced an antibody response against HIV! (December 3, 2022)	18,109; fourth	3906; third

^a^Ranks beyond the fifth were not indicated.

### Anti–HIV Vaccine Conspiracy Theories

RQ 5 asked about prominent anti–HIV vaccine conspiracy theories. Of the 1000 negative posts that received the most reactions, 227 (22.7%) contained conspiracy theories. As Table 7 shows, we classified these prominent anti–HIV vaccine conspiracy theories into 4 categories and presented their representative posts and the number of reactions.

The first category, comprising 44.9% (102/227) of the posts, formulated conspiracy theories by connecting COVID-19, COVID-19 vaccines, HIV, and HIV vaccines. For instance, 52.9% (54/102) of these posts connected the misinformation regarding COVID-19 vaccines containing, causing, or increasing HIV with the ongoing efforts to develop HIV vaccines. This misinformation may have arisen from past occurrences resurfacing following Moderna’s initiation of its mRNA HIV vaccine trials. One incident occurred at the end of 2020, when an Australian COVID-19 vaccine, which used a small fragment of protein from HIV to clamp SARS-CoV-2’s spike proteins, was abandoned due to false HIV-positive results [70]. Another incident occurred in October 2020, when 4 researchers sent a letter to a medical journal expressing concerns about the potential increased risk of HIV acquisition among men receiving COVID-19 vaccines using adenovirus type-5 vectors without supporting data from COVID-19 vaccines [71]. The misinformation typically interpreted the incidents out of context and generally suggested that COVID-19 vaccines contained, caused, or increased HIV without specifying details. In addition, there were conspiracy theories linking HIV and AIDS to COVID-19 vaccine side effects, including a fabricated condition known as VAIDS. VAIDS falsely suggests that COVID-19 vaccines caused immune deficiency [72]. Furthermore, there were claims that COVID-19 originated from unsuccessful HIV vaccine research.

The second category, comprising 38.3% (87/227) of the posts, suggested that the alignment of concurrent events with Moderna’s start of mRNA HIV vaccine trials in late January 2022 was intentional to manipulate the market for HIV vaccines. These events included the rising HIV discussion and fear; promotion of HIV tests by public figures [73]; the discovery of a new highly virulent HIV strain [74]; and the passing away of HIV researchers, including Luc Montagnier, codiscoverer of HIV with an antivaccine stance during the COVID-19 pandemic [75], all occurring in early February 2022.

The third category, with 11.5% (26/227) of the posts, revealed conspiracy theories based on the distrust of powerholders [76]. Some posts extended existing conspiracy theories, such as the Big Pharma conspiracy theory [50] and the New World Order conspiracy theory [51], into the context of HIV vaccines, emphasizing the intent of powerholders, including major pharmaceutical companies and governments, behind vaccine promotion for financial profits and society control. Other posts created conspiracy theories about the government’s research on HIV vaccines. The remaining posts generally stated that HIV vaccines were a scam. The final category comprised the remaining 5.3% (12/227) of the posts with other conspiracy theories.

It is worth noting that, of the 227 posts containing conspiracy theories, 39 (17.2%) were posted by accounts that had already been suspended at the time of manual coding. For these posts, the X platform displays the following message—“This post is from a suspended account”—and the content of the post is not visible. The X platform suspends accounts that violate its rules [77]. However, specific details of the violations are not accessible on the platform. The invisibility of these posts halted their spread when the suspension was enacted. For our manual coding of these posts, we used the text obtained during the data collection process.

**Table 7:** Anti–HIV vaccine conspiracy theories and representative posts with a high number of reactions (N=227).

Conspiracy theory			Post count, n (%)		Paraphrased posts with a high number of reactions for each conspiracy theory		Reaction count^a^ (likes and reposts)
**HIV and COVID-** **19 (n=102)**							
	Linked to COVID-19 vaccines containing, causing, or increasing HIV	54 (52.9)		The COVID vaccine contained a spike protein derived from HIV. I was banned for saying this and ridiculed for months. Also, pharmacies stock up HIV self-tests. (February 8, 2022)^b^		195 (147 and 48)	
	Linked to whether HIV or COVID-19 vaccines cause injuries such as VAIDS^c^	38 (37.3)		Not about HIV or AIDS. It’s deliberate immune system destruction called VAIDS, Vaccine Acquired Immunodeficiency Syndrome, engineered into vaccines before the pandemic by Gates and Fauci. Coordinated genocide. Stop. (February 12, 2022)^b^		992 (617 and 375)	
	HIV vaccine research causing COVID-19	10 (9.8)		Not surprisingly, this leads to the theory that SARS-CoV-2 was developed as vaccine, possibly against HIV. And it may have leaked. (September 20, 2022)		132 (96 and 36)	
**Linked to HIV-related events (n=87)**							
	Rising HIV discussion and fear	29 (33.3)		Are Canadians overlooking the connection between recent media coverage on an HIV epidemic, development of a new HIV vaccine, and their last vaccines? (February 7, 2022)		7766 (5887 and 1879)	
	HIV tests urged by public figures	28 (32.2)		Three Members of Parliament announced their recent HIV test results in the past few days. Oxford University is developing an HIV vaccine. When will HIV vaccines become mandatory? (February 7, 2022)		1182 (890 and 292)	
	New highly virulent HIV strain found	20 (23)		By coincidence again, the development of a new mRNA HIV vaccine began just before the emergence of the new HIV strain. (February 8, 2022)		106 (102 and 4)	
	HIV researchers passing away	10 (11.5)		Prominent HIV researchers are facing assassinations. Why are they being targeted after data shows Covid vaccine causes HIV symptoms? There is an effort to silence those with revealing knowledge. (February 10, 2022)		630 (501 and 129)	
**Powerholders’ interests (n=26)**							
	Big Pharma’s profits	6 (23.1)		Big Pharma stages a fake pandemic and profits from drugs. They add HIV to drugs to trigger an AIDS crisis and then promote an HIV vaccine. (February 8, 2022)		23 (16 and 7)	
	Depopulation and New World Order	4 (15.4)		How can we reclaim our nation from destructive forces and establish a New World Order? Covid hoax, HIV vaccine depopulation, staged riots, election manipulation. WEF destroys countries globally. (December 16, 2022)		23 (14 and 9)	
	Linked to government HIV vaccine research	8 (30.8)		The highly revered person is one accountable. If he could do it then, what did he do with mRNA? Revealed: How vulnerable children was treated with hardship in Fauci’s obsessive pursuit of an HIV vaccine? (December 21, 2022)		90 (59 and 31)	
	HIV vaccine as a scam	8 (30.8)		I sense the emergency of an HIV vaccine scam. (February 9, 2022)		588 (518 and 70)	
Other			12 (5.3)		—^d^		—

^a^The reaction count is the total number of likes and reposts.

^b^The posts were from suspended accounts.

^c^VAIDS: vaccine-acquired immunodeficiency syndrome.

^d^The categories labeled as “other” contain various conspiracy theories. Thus, no representative post is displayed.

## Discussion

### Principal Findings

This study investigated the patterns of public discourse and the message-level drivers of user reactions on the X platform regarding HIV vaccines through the analysis of posts using machine learning algorithms. We examined the distribution of topics and valence across different post types and assessed the influence of content features (topics and valence) and account features (account verification status and follower count) on like and repost counts. In addition, we manually coded the 1000 most engaged posts about HIV and COVID-19 to understand the salient aspects of HIV vaccines related to COVID-19 and the 1000 most engaged negative posts to identify prominent anti–HIV vaccine conspiracy theories.

The results revealed that COVID-19 plays a substantial role as a context for public discourse and reactions regarding HIV vaccines. Of the 3 topics identified using LDA, the leading topic was HIV and COVID-19, covering 78% of tokens and dominating in 92.46% (33,678/36,424) of the posts. Furthermore, on each of the top 4 days with the highest post counts, most of the posts were about HIV and COVID-19. This comprehensive topic included important subtopics that linked HIV vaccines with COVID-19 vaccines, as demonstrated through the manual coding of the 1000 most engaged posts about HIV and COVID-19. These subtopics encompassed the reciprocal influence of HIV vaccines and COVID-19 vaccines in advancing each other’s development; comparisons in their development speed; inquiries about the possible alignment of HIV vaccines with COVID-19 vaccines in terms of cost and accessibility during distribution; and concerns about efficacy, safety, and equality for both vaccines.

COVID-19 positioned HIV vaccines in both a positive and negative context. On the one hand, the success of mRNA technology in COVID-19 vaccines [6] potentially cast mRNA HIV vaccines in a positive light. The topic of HIV and COVID-19 had a marginally positive valence score of 0.055. Moreover, 3 (60%) out of the 5 most liked posts and 2 (40%) out of the 5 most reposted posts expressed excitement about advancements in HIV vaccines that were based on the experience with COVID-19 vaccines. On the other hand, antivaccine discourse, including conspiracy theories, heated up during the COVID-19 pandemic [10,11,27,44,48,49], which posed challenges to HIV vaccines. Of the 1000 most engaged posts about HIV and COVID-19, a total of 136 (13.6%) featured conspiracy theories. Of the 1000 most engaged negative posts, 227 (22.7%) contained conspiracy theories, with 102 (44.9%) of them revolving around HIV and COVID-19. For instance, a prominent conspiracy theory connected the misinformation about COVID-19 vaccines containing, causing, or increasing HIV infection [55] with the initiation of clinical trials for mRNA HIV vaccines [4,5], implying a malevolent intent behind the deliberate connection. The results indicate that conspiracy theories tend to elicit an approach-oriented response, as evidenced by people engaging in liking and reposting, as opposed to an avoidance-oriented approach [39]. This underscores the need to intensify efforts to counter conspiracy theories in public health communication about HIV vaccines.

According to a study conducted by the Pew Research Center, irrespective of the subject matter, replies constituted the largest portion of original posts on X, followed by self-composed and quote posts [28]. Specifically, the number of replies was 3 times greater than that of self-composed posts. In this study, although replies constituted slightly more than half (18,580/36,424, 51.01%) of the posts, it is worth noting that the subject of HIV vaccines elicited a higher proportion of self-composed posts at 41.6% (15,151/36,424). Specifically, the number of replies was 23% higher than that of self-composed posts. Moreover, the topic of mRNA vaccine trials was most evident in self-composed posts compared to replies and quote posts. In comparison, there was a higher proportion of focus on the topic of HIV and COVID-19 and a greater proportion of negative posts among quote posts and replies, which contribute to existing conversations. This suggests that users were more likely to initiate new conversations rather than joining existing conversations about mRNA HIV vaccines. In contrast, they were more likely to join existing conversations rather than starting new conversations about HIV and COVID-19. In addition, users were less likely to initiate new conversations negatively but more likely to contribute negatively to existing ones.

As the primary topic, HIV and COVID-19 had no impact on like counts but had a negative impact on repost counts. In comparison, the topic of mRNA HIV vaccine trials had a negative impact on like counts and a positive impact on repost counts. The results should be interpreted while considering that, as revealed in previous research [16,34] and this study, most posts on the X platform are unlikely to receive likes and even less likely to receive reposts. In this study, among the total of 36,424 posts, approximately half (n=19,284, 52.94%) received likes, and approximately one-quarter (n=9155, 25.13%) received reposts. To include all posts and mitigate the data distribution skewness in the linear regression analysis, we applied the “plus one” technique. This involved adding a constant value of 1 to all like and repost data points before taking the natural logarithm. Although most posts were not liked or reposted, it is noteworthy that the topic of mRNA HIV vaccines led to an increase in repost counts, highlighting its positive influence on social sharing. In addition, 2 (40%) out of the 5 most reposted posts were about mRNA HIV vaccine trials. These results correspond to the findings of previous research that suggested the diffusion of novel useful information [12,16,32,36].

The overall valence of the posts about HIV vaccines was marginally positive. The positivity aligns with the positive sentiment found in posts on X about vaccines in general [13-15] and COVID-19 vaccines in particular [12,16,17]. However, the positivity about HIV vaccines was not apparent as the average score of 0.053 placed it on the edge of the neutral range, which goes from −0.05 to 0.05 according to the standard VADER classification values. Positive sentiment had a favorable impact on like and repost counts, partially consistent with findings of previous research on COVID-19 vaccines [16]. The post that achieved the most likes conveyed the incredible feeling of witnessing the development of an HIV vaccine in our lifetimes. This could be attributed to the psychological rationale that social transmission of positive content fulfills people’s motivation to present a positive image [35,37]. In alignment with the findings of previous research [13,16,34], account verification status and follower count increased like and repost counts.

This study has implications for public health communication related to HIV vaccines and potentially other vaccines. Given the massive scale of the COVID-19 vaccination campaign, it is understandable that people will draw comparisons with other vaccines. Topic modeling identified HIV and COVID-19 as the primary topic, and manual coding revealed various intertwined aspects. Leveraging the advantages observed in the COVID-19 vaccine campaign, such as its widespread accessibility, could be valuable. Furthermore, addressing common concerns such as efficacy, safety, and inequality could also prove beneficial.

In the case of HIV vaccines, it is essential to tackle concerns associated with COVID-19 vaccines, especially those related to HIV vaccines. A major subtopic of HIV and COVID-19 involved suspicions about COVID-19 vaccines containing, causing, or increasing HIV. Another major subtopic was the confusion between HIV symptoms and the alleged side effects of COVID-19 vaccines, such as VAIDS. Misinformation concerning both subtopics has been woven into conspiracy theories, further complicating this situation. To combat misinformation and conspiracies that have these elements, efforts could focus on promoting evidence-based factual information [45-47].

Another notable technique in the conspiracy theories was linking concurrent COVID-19 and other HIV-related events in unsubstantiated relationships to create false perceptions, suggesting that these events were intentional to manipulate the market for HIV vaccines. These HIV-related events included rising HIV discussion and fear, promotion of HIV tests by public figures [73], the discovery of a new highly virulent HIV strain [74], and the passing away of HIV researchers, all occurring in early February 2022. These findings suggest that refuting false connections among such concurrent events can be an effective strategy to counter these conspiracy theories [45-47]. These occurrences, frequently entwined within conspiracy theories, could be specifically addressed in public health communication efforts.

### Limitations

This study has several limitations. Because we used autoidentified content features (topics and valence) and autoextracted account features (verification status and follower count) in the regression models to predict the autoextracted number of user reactions (likes and reposts), the results were mostly limited to the examined autoidentified and autoextracted factors. For instance, political polarization, which manifested in a wide range of issues, including response to vaccines [78], could be a factor worth investigating in future studies. Furthermore, manual coding of conspiracy theories revealed a prevalent technique of twisting concurrent events into false relationships. This underscores the significance of refuting unfounded associations among these incidents to counter such conspiracy theories. It will be interesting for future research to assess the impact of this technique on user reactions to conspiracy theories. These findings could provide further insights into public health communication strategies to combat conspiracy theories.

### Conclusions

The results highlight COVID-19 as a significant backdrop for public discourse and reactions on the X platform regarding HIV vaccines. COVID-19 situated HIV vaccines in both a positive and negative context. The success of mRNA COVID-19 vaccines shed a positive light on HIV vaccines. However, COVID-19 also situated HIV vaccines in a negative context, as evident in anti–HIV vaccine conspiracy theories falsely linking HIV vaccines to COVID-19. The findings provide implications for public health communication strategies concerning HIV vaccines.

## Abbreviations

LDA: latent Dirichlet allocation
mRNA: messenger RNA
RQ: research question
VADER: Valence Aware Dictionary and Sentiment Reasoner
VAIDS: vaccine-acquired immunodeficiency syndrome

## Data Availability

The data sets collected and analyzed during this study are available from the corresponding author upon request.
